# Characterizing *Aeromonas* spp. as a Potential Sentinel Organism for Antimicrobial Resistance Dissemination in Wastewater and Drinking Water Treatment Systems: A Case Study in the Barcelona Metropolitan Area, Spain

**DOI:** 10.3390/antibiotics15030301

**Published:** 2026-03-17

**Authors:** Laura Mondéjar, Victoria Ballén, Yaiza Gabasa, Laura Castellsagués, Anna Pinar-Méndez, Carles Vilaró, Belén Galofré, Aida González-Díaz, Sara Martí, Sergi Sanz, Sara M. Soto

**Affiliations:** 1 Barcelona Institute for Global Health (ISGlobal), 08028 Barcelona, Spain; laura.mondejar@isglobal.org (L.M.); vickyballen@gmail.com (V.B.); yaiza.gabasa@isglobal.org (Y.G.); laura.castellsagues.florit.99@gmail.com (L.C.); sergi.sanz@isglobal.org (S.S.); 2 Faculty of Pharmacy and Food Sciences, University of Barcelona (UB), 08028 Barcelona, Spain; 3 Aigües de Barcelona, Empresa Metropolitana de Gestió del Cicle Integral de l’Aigua, 08028 Barcelona, Spain; anna.pinar@aiguesdebarcelona.cat (A.P.-M.); carles.vilaro@aiguesdebarcelona.cat (C.V.); belen.galofre@aiguesdebarcelona.cat (B.G.); 4 Microbiology Department, Hospital Universitari Bellvitge, IDIBELL-UB, 08907 L’Hospitalet de Llobregat, Spain; agonzalezd@bellvitgehospital.cat (A.G.-D.); smartinm@bellvitgehospital.cat (S.M.); 5 Consorcio de Investigación Biomédica en Red de Enfermedades Respiratorias (CIBERES), 08950 Madrid, Spain; 6 Consorcio de Investigación Biomédica en Red de Epidemiología y Salud Pública (CIBERESP), 08950 Madrid, Spain; 7 Faculty of Medicine and Health Sciences, University of Barcelona (UB), 08007 Barcelona, Spain; 8 CIBER Enfermedades Infecciosas (CIBERINFEC), Instituto de Salud Carlos III, 28029 Madrid, Spain

**Keywords:** *Aeromonas* spp., antimicrobial resistance, antibiotic resistance genes, wastewater treatment plants, drinking water treatment, horizontal gene transfer, biofilms, environmental surveillance

## Abstract

**Background:** Wastewater treatment plants (WWTPs) are hotspots of antimicrobial resistance (AMR) due to inputs from diverse anthropogenic sources. *Aeromonas* spp., ubiquitous in aquatic environments, often carry clinically relevant antibiotic resistance genes (ARGs) and can persist beyond fecal contamination indicators, making them promising sentinel organisms for AMR dissemination. The aim of this study was to assess the suitability of *Aeromonas* spp. in this role by characterizing resistance profiles, associated virulence factor genes (VFGs), genetic mobility, and persistence across wastewater and drinking water treatment processes in the Barcelona metropolitan area, Spain. **Methods**: Isolates were phenotypically characterized and screened for ARGs, VFGs, integrons, and heavy metal tolerance genes, followed by whole-genome sequencing (WGS). Biofilm formation was assessed in vitro. Conjugation assays with *Escherichia coli* evaluated horizontal gene transfer (HGT) potential. **Results:** A total of 428 antibiotic-resistant *Aeromonas* spp., the most abundant antibiotic-resistant bacteria isolated during the 2023 sampling campaigns from two WWTPs and one drinking water treatment plant (DWTP), were characterized. Trimethoprim/sulfamethoxazole (SXT) non-susceptibility was most frequent (72%), followed by cefoxitin resistance (65.4%). The *sul1* (57.5%) and *bla*_MOX_ (78.6%) genes predominated among SXT- and β-lactam-resistant isolates. The *merA* gene was detected in 23.6%; 97.9% harbored at least one VFG (*aerA*, *act*, *fla*, *alt*, or *hlyA*), and 70.3% carried *intI1*. Half formed biofilm. Conjugation confirmed bi-directional HGT, and WGS revealed persistent ST3458 clones across treatment stages. **Conclusions:** WWTPs and DWTPs act as reservoirs of antibiotic-resistant *Aeromonas* spp., demonstrating persistence and HGT potential. Findings support their use as sentinel organisms for AMR surveillance in aquatic environments and for assessing treatment efficacy, highlighting variability across treatment types and locations, and reinforcing their relevance for urban water reclamation monitoring.

## 1. Introduction

Antimicrobial resistance (AMR) has become a critical global health threat, undermining antibiotic effectiveness and posing significant risks to public health, agriculture, and ecosystems [1]. Beyond clinical settings, the environment, particularly aquatic ecosystems, plays a crucial role in the maintenance and dissemination of AMR [2,3]. The urban water cycle, encompassing wastewater treatment plants (WWTPs), drinking water treatment plants (DWTPs), and surface waters, acts as a complex interface where antibiotic-resistant bacteria (ARB) and antibiotic-resistance genes (ARGs) can circulate, persist, and spread [2,4].

WWTPs are designed to eliminate suspended solids, biodegradable compounds, and excess nutrients from sewage using a combination of physical, chemical, and biological methods before returning it to environmental ecosystems [5]. However, their capacity to remove ARB and ARGs remains insufficiently characterized [6]. When treated wastewater is subsequently reused, it can become a likely route for the dissemination of AMR [7]. The new European directive on wastewater treatment (Directive (EU) 2024/3019) [8] only requires monitoring of antibiotic resistance at the inlet of WWTPs, without any parametric value.

DWTPs are primarily designed to make water completely safe for human consumption. Depending on many factors, such as the source of the water, its availability, the size of the population to be served, etc., DWTPs may have a wide variety of treatment stages with the goal of eliminating any chemical or microbiological risks for people, meeting the quality and safety levels required by different drinking water regulations. The current European Drinking Water Directive (Directive (EU) 2020/2184) [9] does not specify limit values for ARB or ARGs, and DWTPs are not specifically designed for their removal. Their monitoring is at the operator’s discretion. If ARB and ARGs are present in the source water entering into DWTPs, this could inadvertently enhance the survival and spread of ARB and encourage horizontal gene transfer (HGT), which could pose health threats to the population, making their reduction in these systems a priority [10,11].

Among the diverse bacterial taxa found in water systems, *Aeromonas* spp. are of particular concern [12]. These Gram-negative, facultative anaerobic organisms are ubiquitous in aquatic environments and include both environmental and opportunistic pathogenic species [13]. Among the clinically relevant opportunistic pathogens, *A. hydrophila*, *A. caviae*, *A. veronii*, and *A. dhakensis* are capable of causing a broad spectrum of gastrointestinal, bloodstream, skin, soft tissue, abdominal, respiratory, and urogenital infections, as well as ocular diseases [14]. Other *Aeromonas* species are primarily environmental, with limited or no reported human infections, but can still act as reservoirs for ARGs [15]. Their detection in treated and untreated water [5,16] underscores their dual relevance as both environmental organisms and indicators of potential clinical AMR dissemination [17].

Recent studies have proposed environmental bacteria, including *Aeromonas* spp., as biomarkers for AMR monitoring across aquatic systems [18]. This approach becomes especially pertinent in the context of the One Health framework, which highlights the interconnectedness of human, animal, and environmental health in combating AMR [19].

Beyond ARGs, mobile genetic elements (MGEs), including integrons and plasmids, together with heavy metal tolerance genes (HMTGs) and virulence factor genes (VFGs), are valuable indicators for understanding how AMR spreads in the environment [20]. VFGs enhance pathogenicity by facilitating host colonization, immune evasion, and tissue damage and may act synergistically with ARGs, thereby increasing the clinical impact of infections [21]. Similarly, biofilm formation may enhance bacterial persistence in aquatic environments [22]. Moreover, conjugation demonstrates HGT, further reinforcing the environmental concerns linked to *Aeromonas* spp. [23].

Although the spread of AMR is a serious and escalating collective health issue, efforts to track its progression in environmental settings remain limited [24]. Understanding how these bacteria evolve and persist in such interconnected systems is crucial for developing early-warning tools and improving water management strategies.

The aim of this study was to characterize antibiotic-resistant *Aeromonas* spp. isolated across different treatment stages from two WWTPs located in Baix Llobregat and Gavà, and one DWTP located in Sant Joan Despí, with all three belonging to the metropolitan area of Barcelona, Spain. We investigated their antimicrobial susceptibility profiles, ARGs, VFGs, integrons, HMTGs, biofilm-forming ability, and conjugative capacity to assess their suitability as biomarkers of AMR in aquatic environments.

## 2. Results

### 2.1. Identification of Aeromonas spp. Isolates

In a previous study [25] conducted in our laboratory, 991 ARBs were isolated from two WWTPs and one DWTP, with *Aeromonas* being the most prevalent genus, accounting for 439 isolates (44.3%). Whole-genome sequencing revealed 11 genetically identical isolates, which were excluded from this investigation, resulting in a final set of 428 *Aeromonas* spp. (43.7%). Of these, 230 (53.7%) were collected from the Baix Llobregat WWTP, 180 (42.1%) from the Gavà-Viladecans WWTP, and 18 (4.2%) from the inlet of the Sant Joan Despí DWTP. No *Aeromonas* spp. isolates were detected at the outlet of the DWTP.

Species-level identification was performed for 208 isolates (48.6% of all *Aeromonas*) comprising the following: *A. caviae* (17.7%, *n* = 76), *A. veronii* (11.9%, *n* = 51), *A. hydrophila* (6.3%, *n* = 26), *A. media* (3.7%, *n* = 16), *A. jandaei* (2.1%, *n* = 9), *A. allosaccharophila*, *A. rivipollensis* and *A. eucrenophila* with five isolates each (1.2%, *n* = 5), *A. sanarellii* (0.9%, *n* = 4), *A. bestiarum* and *A. sobria* with three isolates each (0.7%), *A. salmonicida* (0.4%, *n* = 2), and *A. encheleia*, *A. simiae* and *A. taiwanensis* with one isolate each (0.2%). The remaining 220 isolates (51.4%) that could not be reliably identified at the species level were classified as *Aeromonas* spp., due to database limitations.

### 2.2. Impact of Treatment Stages on Aeromonas spp. Prevalence in WWTPs and DWTP

At the Baix Llobregat WWTP, the number of *Aeromonas* spp. decreased from 83 isolates (36%) in the primary inlet to 59 (25.7%) in the secondary outlet. However, a moderate increase was detected at the tertiary outlet, with 73 isolates (31.8%) being collected. After the advanced tertiary stage, a marked reduction occurred (*n* = 15, 6.5%), highlighting the effectiveness of the final treatment in bacterial removal. On the other hand, the Gavà-Viladecans WWTP showed a progressive decrease from 82 isolates (45.6%) in the primary inlet to 61 (33.8%) in the integrated fixed-film activated sludge (IFAS) secondary outlet, followed by reductions to 31 isolates (17.3%) at the advanced membrane bioreactor (MBR) secondary outlet, and six (3.3%) in the tertiary outlet. This pattern demonstrates the cumulative effect of each treatment stage in reducing the *Aeromonas* spp. population. Finally, the Sant Joan Despí DWTP effectively removed the 18 (100%) *Aeromonas* spp. detected at the inlet, resulting in their complete absence in the final drinking water.

### 2.3. Antimicrobial Resistance Profiles and Classification

**Figure 1:**
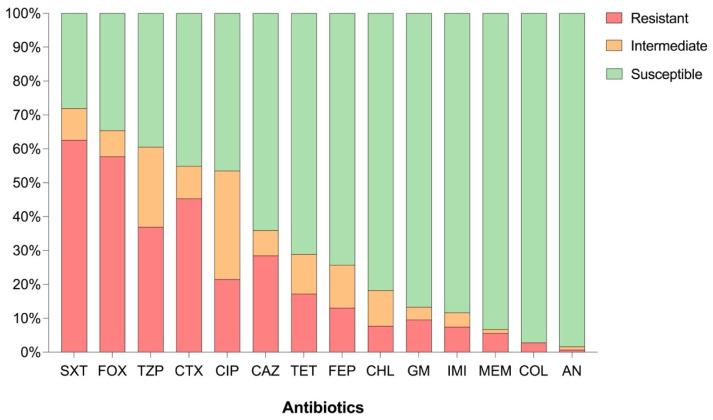
Percentage of *Aeromonas* spp. isolates classified as resistant, intermediate or susceptible to the different antibiotics. AN: amikacin, CAZ: ceftazidime, CHL: chloramphenicol, CIP: ciprofloxacin, COL: colistin, CTX: cefotaxime, FEP: cefepime, FOX: cefoxitin, GM: gentamicin, IMI: imipenem, MEM: meropenem, SXT: trimethoprim/sulfamethoxazole, TET: tetracycline, TZP: piperacillin–tazobactam.

The AMR profiles of the *Aeromonas* spp. isolates were consistent across all three treatment plants. Over 70% of the isolates (*n* = 308) exhibited intermediate susceptibility or resistance to trimethoprim/sulfamethoxazole (SXT), while 65.4% were non-susceptible to cefoxitin (FOX) (*n* = 280), and 60.7% (*n* = 260) were non-susceptible to piperacillin–tazobactam (TZP). A considerable proportion of isolates also showed intermediate susceptibility or resistance to cefotaxime (CTX) and ciprofloxacin (CIP), with rates of 54.9% (*n* = 235) and 53.5% (*n* = 229), respectively. Non-susceptibility to ceftazidime (CAZ) was observed in 35.9% of the isolates (*n* = 154). Lower non-susceptibility rates were observed for tetracycline (TET) (28.9%, *n* = 124), cefepime (FEP) (25.7%, *n* = 110), chloramphenicol (CHL) (18.2%, *n* = 78), gentamicin (GM) (13.3%, *n* = 57), imipenem (IMI) (11.4%, *n* = 49), meropenem (MEM) (7%, *n* = 30), colistin (COL) (2.8%, *n* = 12), and amikacin (AN) (1.6%, *n* = 7) (Figure 1).

**Figure 2:**
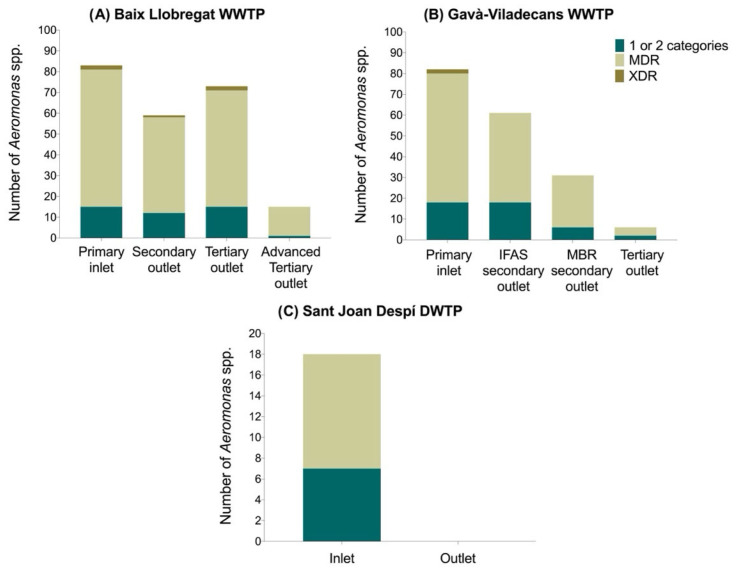
Distribution and AMR of *Aeromonas* spp. isolates across treatment stages in the treatment plants studied. (**A**) Baix Llobregat WWTP; (**B**) Gavà-Viladecans WWTP; (**C**) Sant Joan Despí DWTP.

A total of 94 isolates (22%) displayed resistance to one or two antimicrobial categories, 327 isolates (76.4%) were classified as multidrug resistant (MDR), and 7 isolates (1.6%) as extensively drug-resistant (XDR). Although their numbers decreased from the initial to the final stage, MDR isolates and those non-susceptible to one or two antimicrobial classes were detected at all water treatment stages in both WWTPs (Figure 2A, B) and at the DWTP inlet (Figure 2C). XDR *Aeromonas* spp. isolates were detected up to the tertiary treatment stage in the Baix Llobregat WWTP (Primary inlet, *n* = 2; Secondary outlet, *n* = 1; Tertiary outlet, *n* = 2). In the Gavà-Viladecans WWTP, XDR isolates were present only in the primary inlet (*n* = 2). Notably, no XDR isolates were detected at the DWTP inlet.

It is worth noting that although these three resistance categories significantly reduced after the final treatment stage at the Baix Llobregat WWTP, an increase was observed between the secondary outlet and the tertiary outlet: isolates resistant to one or two categories rose from 12 to 15, MDR isolates from 46 to 56, and XDR isolates from one to two.

### 2.4. Phenotypic Characterization of Extended-Spectrum β-Lactamase (ESBL), AmpC and Carbapenemase-Producing *Aeromonas* spp. Isolates

Phenotypic screening for ESBL and AmpC production was conducted in 240 isolates exhibiting non-susceptibility to third-generation cephalosporins (3GC). Among these, 153 isolates were identified as ESBL producers (63.7%), with 24 harboring CTX-M group enzymes as determined by the NG-Test^®^/CTX-M Multi immunochromatographic assay (NG-Biotech Laboratories, Guipry, France). Additionally, 131 (54.6%) isolates were identified as AmpC producers.

Among the 54 isolates exhibiting non-susceptibility to carbapenems, the modified carbapenem inactivation method (mCIM) was performed, followed by the EDTA-carbapenem inactivation method (eCIM). Forty isolates tested positive by mCIM; of these, 29 were also positive by eCIM, indicating the presence of a metallo-β-lactamase. These 40 isolates were further analyzed using the NG-Test^®^ DetecTool CARBA 5 (NG-Biotech Laboratories, Guipry, France), which identified 29 positive isolates: 17 as KPC producers, four as VIM producers and eight co-carrying both KPC and VIM enzymes.

### 2.5. Antimicrobial Resistance Genes (ARGs)

**Figure 3:**
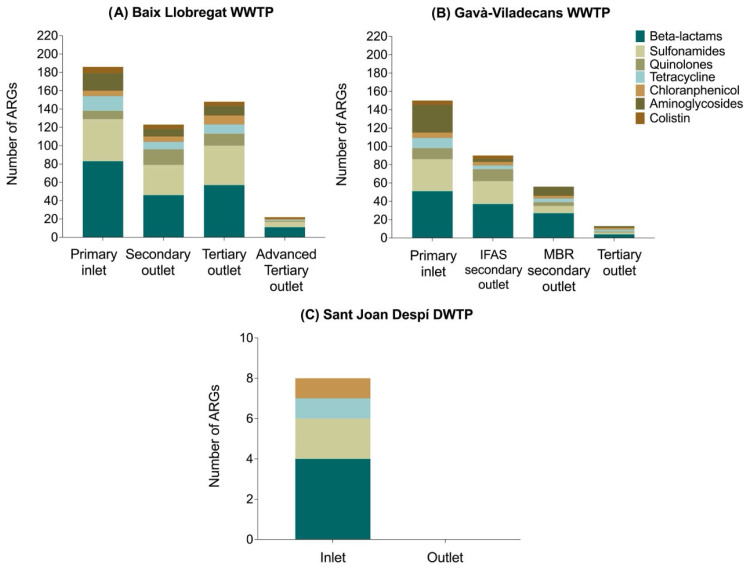
Distribution of ARGs detected in *Aeromonas* spp. across treatment stages in the treatment plants. (**A**) Baix Llobregat WWTP; (**B**) Gavà-Viladecans WWTP; (**C**) Sant Joan Despí DWTP.

The *Aeromonas* spp. isolates analyzed in both WWTPs carried a wide diversity of ARGs, including genes conferring resistance to β-lactams, sulfonamides, quinolones, tetracyclines, chloramphenicol, aminoglycosides, and colistin. In contrast, the Sant Joan Despí DWTP inlet presented isolates carrying less diverse ARGs, with no detection of quinolone, aminoglycoside, or colistin genes (Figure 3).

Within the *Aeromonas* spp. isolates phenotypically identified as ESBL producers (*n* = 153), 16 isolates only carried the *bla*_CTX-M-1_ group genes, while one isolate carried a *bla*_CTX-M-9_ group gene. In addition, 30 isolates only harbored the *bla*_VEB_, 21 *bla*_PER_, 12 *bla*_TEM_, three *bla*_SHV_ genes, and two *bla*_GES_ genes. Four isolates simultaneously co-carried the *bla*_PER_/*bla*_GES_ genes, three carried both the *bla*_CTX-M-1_/*bla*_TEM_ genes, and two harbored both *bla*_PER_/*bla*_VEB_ genes. Additionally, two isolates harbored the *bla*_TEM_/*bla*_VEB_ genes, one was positive to both *bla*_CTX-M-1_/*bla*_VEB_ genes, one carried the *bla*_TEM_/*bla*_PER_ gene combination, and one isolate harboring the gene combination *bla*_SHV_/*bla*_PER_ was detected. Lastly, three bacteria were simultaneously positive for the *bla*_CTX-M-1_/*bla*_TEM_/*bla*_VEB_ genes.

Regarding the isolates presenting a phenotypic AmpC profile (*n* = 131), we detected 95 only harboring the *bla*_MOX_, 14 carrying the *bla*_FOX_, and seven carrying the *bla*_CIT_ gene. Eight isolates co-harbored two or more AmpC genes: four harbored the combination of *bla*_MOX_/*bla*_CIT_ genes and four both *bla*_MOX_/*bla*_FOX_ genes. No *bla*_DHA_ or *bla*_EBC_ genes were found.

Besides the 29 carbapenemase-producing isolates detected by the NG-Test^®^ DetecTool CARBA 5, 25 isolates carried only the *bla*_CPHA_ gene, one *bla*_KPC-_positive isolate also harbored the *bla*_CPHA_ gene, and another isolate simultaneously co-carried the *bla*_KPC_/*bla*_VIM_/*bla*_CPHA_ genes.

The subset of isolates non-susceptible to CIP (*n* = 229) was tested for the *qnrA*, *qnrB*, *qnrC and qnrS* genes. Only the *qnrS* gene was detected in 70 isolates.

Among the 60 aminoglycoside-resistant or intermediate isolates, nine harbored the *aac(6′)-lb-cr* gene and one carried the *aadA5* gene. Thirteen isolates co-carried the *aph(6)-Id/aph(3”)-lb/aac(6′)-lb-cr* genes, four the *aph(6)-Id/aph(3”)-lb* genes, three simultaneously harbored the *aph(6)-Id/aadA5/aph(3”)-lb/aac(6′)-lb-cr* genes, and three isolates presented the *aph(3”)-lb/aac(6′)-lb-cr* genes. Additionally, two isolates were positive for both *aadA5/aac(6′)-lb-cr* genes, one co-carried the *aph(6)-Id/aadA5/aph(3”)-lb* genes, and one the *aph(6)-Id/aac(6′)-lb-cr* genes.

Among the isolates non-susceptible to sulfonamides (*n* = 308), 165 harbored the *sul1* gene, 11 carried the *sul2* gene, and 12 isolates co-carried both genes.

In the group of *Aeromonas* spp. isolates phenotypically non-susceptible to CHL (*n* = 78), eight carried the *catI gene*, 26 harbored the *cmlA gene*, and two isolates co-carried both genes.

Detection of resistance genes in TET-non-susceptible isolates (*n* = 124) revealed the presence of the *tetA* gene in 54 isolates and the *tetB* gene in three isolates.

No *mcr-3* genes were detected by polymerase chain reaction (PCR) among the isolates showing phenotypic resistance to COL (*n* = 12). However, among the sequenced isolates, the *mcr-3*, *mcr-3.17*, and *mcr-3.6* genes were detected, despite the isolates not showing a resistant phenotype. These *mcr-3*-carrying isolates were detected as far as the tertiary treatment stage in the Baix Llobregat WWTP and up to the IFAS secondary outlet in the Gavà-Viladecans WWTP.

### 2.6. Integrase, Heavy Metal Tolerance and Virulence Factor Genes

#### 2.6.1. Integrase Genes

Three hundred one isolates (70.1%) were positive for the class 1 integrase gene (*intI1*). Of these, one isolate from the Gavà-Viladecans WWTP IFAS secondary outlet also harbored the *intI2* gene. The 301 *intI1*-positive isolates were distributed across all treatment stages in both WWTPs (Baix Llobregat WWTP: primary inlet, *n* = 64; secondary outlet, *n* = 43; tertiary outlet, *n* = 46; advanced tertiary outlet, *n* = 10; Gavà-Viladecans WWTP: primary inlet, *n* = 56; IFAS secondary outlet, *n* = 47; MBR secondary outlet, *n* = 24; tertiary outlet, *n* = 5), and in the DWTP inlet (*n* = 6).

#### 2.6.2. Heavy Metal Tolerance Genes (HMTGs)

Regarding the four HMTGs studied (*merA*, *silA*, *pcoD* and *arsA* genes), a total of 93 isolates (21.7%) harbored the *merA* gene (conferring mercury resistance), and eight (1.8%) carried both *merA* and *arsA* genes (the latter conferring arsenic resistance). Additionally, none of the isolates were positive for the *silA* gene (conferring silver resistance) or the *pcoD* gene (copper resistance). As shown in Table 1, the *merA* gene was detected among the *Aeromonas* spp. isolates across all treatment stages of both WWTPs and the DWTP inlet. In contrast, the *arsA* gene was detected at all treatment stages except at the final effluent points. No *arsA*-carrying isolates were detected at the inlet of the DWTP.

#### 2.6.3. Virulence Factor Genes (VFGs)

A total of 419 isolates (97.9%) carried at least one of the following VFGs: *aerA* (aerolysin), *act* (cytotoxic enterotoxin), *fla* (flagellin), *alt* (cytotonic heat-labile enterotoxin), or *hlyA* (hemolysin). Among these, the *act* and *alt* VFGs presented the highest prevalence, being detected in 82.7% (*n* = 354) and 72% (*n* = 308) of the isolates, respectively. In contrast, the prevalence of the remaining VFGs was notably lower, with the *fla gene* identified in 29.4% (*n* = 126), the *aerA* gene in 21.7% (*n* = 93) and the *hlyA* gene in only 3.5% (*n* = 15) of the isolates.

**Figure 4:**
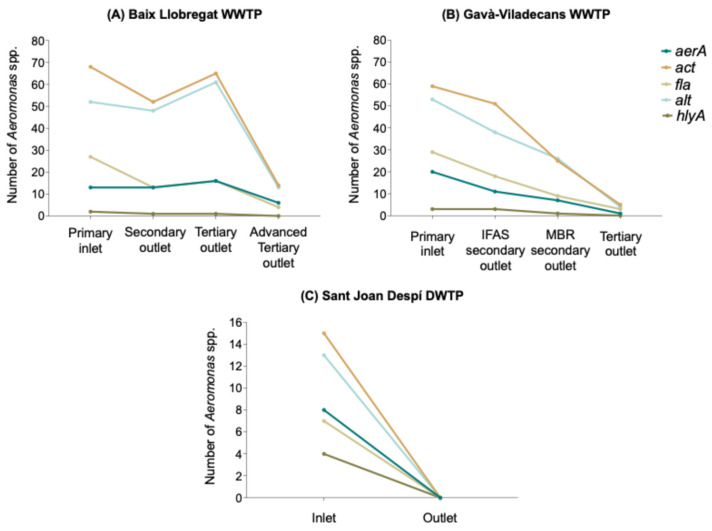
Number of antibiotic-resistant *Aeromonas* spp. carrying each VFG across the treatment stages in WWTPs and the DWTP. (**A**) Baix Llobregat WWTP; (**B**) Gavà-Viladecans WWTP; (**C**) Sant Joan Despí DWTP.

*Aeromonas* spp. carrying the *aerA*, *act*, *fla*, and *alt* genes were detected in all the treatment points of both WWTPs (Figure 4A, B) and the DWTP inlet (Figure 4C), while isolates carrying the *hlyA* gene were absent in the final stages of both WWTPs. All the VFGs analyzed were detected among the *Aeromonas* spp. isolates from the Sant Joan Despí DWTP inlet.

### 2.7. Biofilm-Forming Ability of the Isolates

A total of 37 isolates (8.7%) were considered strong biofilm formers (SBF) (optical density (OD) ≥ 0.817), 71 (16.8%) were classified as moderate biofilm formers (MBF) (0.408 ≤ OD ≤ 0.817), 108 (25.5%) demonstrated weak biofilm formation (0.204 ≤ OD ≤ 0.408), and 207 (49%) did not have the ability to form biofilm (OD ≤ 0.204).

**Figure 5:**
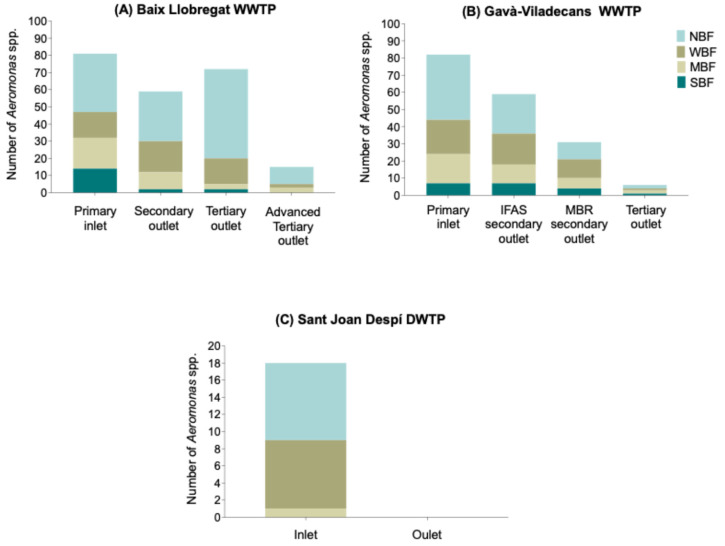
Number of antibiotic-resistant *Aeromonas* spp. isolates classified by biofilm formation category across treatment stages in the treatment plants. (**A**) Baix Llobregat WWTP; (**B**) Gavà-Viladecans WWTP; (**C**) Sant Joan Despí DWTP.

Biofilm-forming isolates were more abundant than non-biofilm formers (NBF) at the primary inlet and secondary outlet of the Baix Llobregat WWTP (Figure 5A), whereas in the Gavà-Viladecans WWTP, this trend persisted across all treatment stages (Figure 5B). On the other hand, at the Sant Joan Despí DWTP inlet, both NBF and biofilm formers were detected in equal numbers (*n* = 9) (Figure 5C).

### 2.8. Horizontal Gene Transfer (HGT)

Due to the fact that *Aeromonas* presented ARGs typical of pathogenic bacteria, the ability to transfer these genes was studied. Firstly, the capacity of HGT from *Aeromonas* spp. to *Escherichia coli* was analyzed by conjugation experiments. The sequenced isolate *A. caviae* 123, carrying an IncW-type replicon and multiple genes (shown in Table 2) was used as a donor. Transconjugant analysis confirmed the transference of the *bla*_PER_, *sul1*, *intI1* and *act* genes from *A. caviae* 123 to *E. coli* CV601. The estimated transfer frequency was 6 × 10^−8^.

To determine whether HGT could occur from *E. coli* to *Aeromonas* spp., a second conjugation experiment was conducted. *E. coli* 1362 strain, containing plasmid replicons FrepB, ColE, and IncU and harboring different ARGs, HMTGs and the class I integrase gene (Table 2), was used as a donor. Two types of transconjugants were detected, one carrying the FrepB replicon marker and another carrying both ColE and IncU replicon markers. PCR analysis of the transconjugants confirmed the transference of *bla*_KPC_ from *E. coli* to *A. caviae* 214 with a transfer frequency of 4.67 × 10^−8^.

Conjugation experiments were performed in triplicate. Transconjugants were confirmed by PCR analysis of selected ARGs and plasmid replicon markers; no additional genomic confirmation was performed.

### 2.9. Whole-Genome Sequencing

All the isolates classified as XDR and a subset of MDR *Aeromonas* spp., all resistant to β-lactams, underwent short-read whole-genome sequencing (*n* = 84). Of these, 59 *Aeromonas* spp. were from the Baix Llobregat WWTP, while 24 isolates were from the Gavà-Viladecans WWTP, and one isolate was from the Sant Joan Despí DWTP.

**Figure 6:**
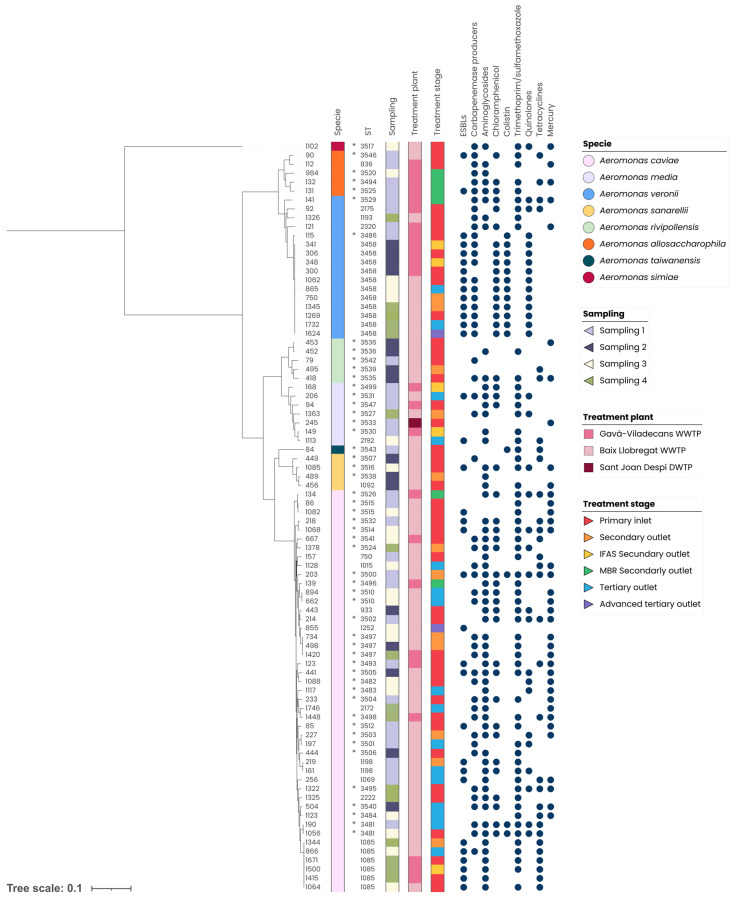
Phylogenetic tree and sequence-type (ST) distribution of *Aeromonas* spp. genomes showing their antibiotic resistance and metal tolerance genes. Species, sampling number, treatment plant and treatment stage were annotated. Blue dots represent the presence of genes conferring resistance to different antibiotic and heavy metal families. IFAS: integrated fixed-film activated sludge, MBR: membrane bioreactor, WWTP: wastewater treatment plant, DWTP: drinking water treatment plant, ESBLs: Extended-spectrum β-lactamases. *: new ST.

These isolates exhibited high genetic diversity based on multilocus sequence typing (MLST), with 62 sequence types (STs) identified, including 46 (74.2%) novel STs. Eight STs comprised multiple isolates: ST3458 (*n* = 11), ST1085 (*n* = 6), ST3497 (*n* = 3), and ST1198, ST3481, ST3510, ST3515, and ST3536 each with two isolates. The remaining STs were represented by a single isolate, as shown in Figure 6.

In addition to β-lactam resistance genes detected in all the sequenced isolates, genes associated with resistance to aminoglycosides, CHL, SXT, COL, quinolones, TET, and mercury tolerance were variably distributed across isolates. Importantly, although phenotypically susceptible, 16 isolates carried ARGs associated with COL resistance; notably, four of these isolates were collected from reclaimed water. It should be noted that an isolate from the secondary outlet of the Baix Llobregat WWTP harbored ARGs conferring resistance to all the antibiotic families analyzed, along with genes associated with mercury tolerance. However, it remained susceptible to CHL, AN, and COL. Moreover, two additional isolates from the Baix Llobregat WWTP lacked genes associated with mercury tolerance but still carried ARGs conferring resistance to all the antibiotic classes tested. Both showed an XDR profile, one showing susceptibility to GM, AN, and COL, and the other to GM and COL.

Three STs (ST3458, ST1085, and ST3497) were markedly more relevant, comprising highly related isolates that were identified across multiple sampling periods, treatment plants, and treatment stages. Although predominantly found in the summer (third sampling, *n* = 3; and fourth sampling, *n* = 4), ST3458 was also detected in the winter (second sampling, *n* = 4). Following a similar pattern, ST3497 was found in the winter (second sampling, *n* = 1) and in the summer (third sampling, *n* = 1; fourth sampling, *n* = 1), whereas ST1085 was exclusively collected during summer samplings (third sampling, *n* = 2; fourth sampling, *n* = 4).

ST3458 was observed at the primary inlet and the IFAS secondary outlet of the Gavà-Viladecans WWTP during the second sampling campaign. In the Baix Llobregat WWTP, this ST was detected at the primary inlet, secondary outlet, and tertiary outlet during the third sampling campaign. Moreover, it persisted across all treatment stages in the fourth sampling, remaining present throughout the entire treatment process. Of note was their presence in the tertiary and the advanced tertiary outlets, both intended for water reuse, and the latter likely connected to the DWTP inlet during drought periods. Interestingly, all the isolates belonging to this ST from the Baix Llobregat WWTP shared identical AMR profiles, harboring genes conferring resistance to β-lactams (*bla*_CTX-M1_, *bla*_OXA-10_, *cphA*), CHL (*cmlA5*), COL (*mcr-3*), and quinolones (*qnrVC4*). Moreover, they harbored *mph(A)* (macrolide resistance) and *qacF* genes (quaternary ammonium tolerance). To further assess the genetic relatedness of ST3458 isolates, a SNP-based phylogenetic analysis was performed using the whole-genome sequencing data. The resulting phylogenetic tree showed that all ST3458 isolates clustered tightly, displaying very short branch lengths and minimal genomic divergence. This SNP-based analysis supports the clonal relatedness of these isolates despite being recovered from different sampling campaigns, treatment stages, and treatment plants (Figure S1).

The isolates assigned to ST3497 were collected from the second and third sampling campaigns at the Baix Llobregat WWTP (both at the secondary outlet), and in the fourth sampling campaign in the Gavà-Viladecans primary inlet. Remarkably, these three isolates exhibited identical AMR profiles, containing ARGs encoding resistance to β-lactams (*bla*_MOX-12_, *bla*_OXA-780_, *bla*_VIM-4_), aminoglycosides (*aph(6)-ld*), sulfonamides (*sul1*), and also showing genes associated with tolerance to mercury (*merR*, *merT*) and quaternary ammonium (*smr*).

On the other hand, ST1085 was restricted to the summer, being identified at the primary inlet and the tertiary outlet of the Baix Llobregat WWTP during the third sampling campaign, and at the secondary outlet during the fourth sampling campaign. At the Gavà-Viladecans WWTP, it was detected in the primary inlet and the IFAS secondary outlet during the fourth sampling campaign. Interestingly, two isolates, one collected from the primary inlet of Gavà-Viladecans WWTP and the other from the secondary outlet of the Baix Llobregat WWTP, shared identical AMR profiles harboring genes associated with resistance to β-lactams (*bla*_CTX-M1_, *bla*_MOX-2_, *bla*_OXA_, *bla*_TEM-1_), aminoglycosides (*aac(3)-lld*), sulfonamides (*sul1*), and TET (*tetE*). Furthermore, they contained *mph(A)* (macrolide resistance).

Isolate 245 (ST3533) from the Sant Joan Despí DWTP inlet was phenotypically resistant to β-lactams, SXT, CIP and TZP, and carried *bla*_OXA_, *bla*_MOX_, and *mer* genes. These genetic determinants make this isolate notable due to its capacity to reach source water for potable supply, posing possible risks to water quality and human exposure.

### 2.10. Associations Among Genetic Determinants and Seasonal Variation

Statistical analyses were conducted to evaluate associations among ARGs, integrase genes, HMTGs, VFGs, and biofilm formation. On the other hand, a seasonal comparison analysis was performed between summer and winter *Aeromonas* isolates. An odds ratio (OR) greater than 1 indicated that the presence of a gene is associated with an increased likelihood of the other gene being present. Some very high OR values were based on small counts of positive observations and should be interpreted with caution, as sparse data can inflate effect estimates even when using Firth’s correction.

Regarding biofilm formation, no positive correlations were found with other genetic determinants, and *intI2* only showed significant correlation (*p* < 0.05) with *bla*_GES_ (OR = 127.91). On the other hand, *intI1* showed associations with *aac(6′)-lb-cr* (OR = 8.04), *mcr-3* (OR = 6.23), *bla*_PER_ (OR = 4.29), and *sul1* (OR = 4.18), and with HMTGs such as *merA* (OR = 2.67).

With respect to HMTGs, both *merA* and *arsA* exhibited possible associations with various ARGs and VFGs. The presence of *merA* was significantly associated with the presence of ARGs such as *catI* (OR = 9.28), *bla*_CIT_ (OR = 4.15), *sul2* (OR = 2.67), *tetA* (OR = 2.37), *bla*_PER_ (OR = 2.35), and *sul1* (OR = 1.74). Moreover, *arsA* tended to occur alongside *sul2* (OR = 9.39), *fla* (OR = 6.48), and *bla*_PER_ (OR = 5.42).

Several significant associations were identified between other VFGs and ARGs. In particular, the *aerA* gene was significantly associated with *bla*_CPHA_ (OR = 9.74), *bla*_FOX_ (OR = 4.48), *bla*_CTX-M-1_ (OR = 3.56), and *bla*_VEB_ (OR = 3.01). The *act* gene was more likely to be present if *bla*_MOX_ (OR = 6.38) or *bla*_KPC_ (OR = 4.18) was present, while the *fla* gene was related to the presence of *bla*_CTX-M-1_ (OR = 4.86). The *alt* gene was significantly associated with *bla*_KPC_ (OR = 6.68), *bla*_MOX_ (OR = 4.06), and *sul1* (OR = 1.85). Finally, the *hlyA* gene had significant associations with *bla*_DHA_ and *bla*_EBC_ (both OR = 87).

Regarding the seasonal comparison, no correlations were found between summer (*n* = 274) and winter isolates (*n* = 154).

## 3. Discussion

The intensive use of antibiotics in human medicine, veterinary care, and agriculture leads to their continuous release into the environment. Numerous studies have identified WWTPs as important reservoirs of AMR [26] and as critical hotspots for the emergence and dissemination of ARB and ARGs [2]. These environmental reservoirs may pose a gateway to DWTPs, particularly under drought conditions, when treated effluents from WWTPs may be used to increase water resources for the production of drinking water [27]. Bacteria capable of colonizing various water resources serve as sentinels for the dissemination of AMR [28]. Among these, the genus *Aeromonas*, characterized by distinct acquired AMR patterns, has emerged as a strong biomarker of environmental AMR [15].

In this study, 428 antibiotic-resistant *Aeromonas* spp. isolates were characterized from two WWTPs and one DWTP in the metropolitan area of Barcelona to evaluate treatment efficacy in removing ARB and ARGs.

In our previous study [25], *Aeromonas* spp. was the most prevalent genus, consistent with other reports [14,29,30,31,32] describing its dominance in wastewater microbial communities. Among the *Aeromonas* species identified, *A. caviae*, *A. veronii*, and *A. hydrophila* were the most prevalent, all of which are well-documented for their pathogenic potential and transmission to humans [31,33].

The *Aeromonas* isolates were similar in both WWTPs, being slightly higher in the Baix Llobregat WWTP. Both plants reduced bacterial loads from the primary inlet to the final outlet. However, a moderate increase in bacterial load was observed at the tertiary outlet of the Baix Llobregat WWTP, whereas the Gavà-Viladecans WWTP showed a consistent decreasing trend across all the treatment stages.

These differences can be attributed to the configuration of these treatment plants as extensively discussed by Ballén et al. [25]. In brief, the tertiary stage at the Baix Llobregat WWTP includes coagulation-flocculation, membrane microfiltration and ultraviolet (UV) disinfection [34]. Coagulation-flocculation may promote bacterial aggregation with the sediment [35], reducing UV penetration [36]. Although microfiltration removes suspended particles, its effectiveness depends on membrane type, integrity, and susceptibility to biofilm formation. UV treatment can also inactivate microorganisms, although its performance is influenced by several operational factors. Moreover, UV light is prone to photoreactivation of surviving bacteria, particularly if sublethal doses are used [37]. In addition, UV disinfection is not combined with chlorine, despite evidence that their synergistic application enhances the removal of ARB and ARGs and limits photoreactivation [38]. By contrast, tertiary treatment at the Gavà-Viladecans facility combines biological treatment with rotary ultrafiltration [39], where physical retention together with shear and friction forces inhibits biofilm formation and disrupts microbial aggregates that may entrap bacteria [40], followed by a final chlorination step. Moreover, in the case of the Baix Llobregat WWTP, an advanced tertiary treatment is implemented specifically for aquifer recharge in the lower Llobregat River basin, with the primary aim of preventing seawater intrusion due to coastal proximity. In contrast, water intended for ecological flow maintenance, agricultural use, and urban irrigation is supplied from the conventional tertiary treatment.

Despite these differences, antibiotic-resistant *Aeromonas* spp. persisted in effluents from both WWTPs, highlighting a possible environmental dissemination of AMR through the final reclaimed water at the WWTP outlet for its reuse.

The *Aeromonas* spp. isolates sequenced in this study belonged to diverse STs, some of which have been previously reported in Spanish WWTPs [5]. The repeated detection of isolates with identical AMR profiles belonging to the same ST over time, across multiple treatment plants and consecutive stages, indicates their potential for spatial dissemination and seasonal adaptation. Specifically, identical ST3458 isolates were present at both the initial and final outlet of the Baix Llobregat WWTP, demonstrating their capacity to survive water treatment processes.

The prevalence of MDR was high (76.4%), in concordance with Harnisz et al. [41], who reported that 76.6% of *Aeromonas* spp. isolated from the municipal wastewater system in Poland were MDR.

In our study, over 70% of the isolates were non-susceptible to SXT, a trend commonly observed in environmental *Aeromonas* isolates, with previous reports [21,42] documenting complete resistance among the isolates tested. Sulfonamide resistance is typically mediated by *sul1* and *sul2*, often carried on class 1 integrons or plasmids, facilitating HGT [43]. In accordance with previous studies [44], our statistical analysis revealed positive correlations between *sul1* and *intI1*.

The CIP resistance observed (53.5%) aligns with previous publications [45] reporting resistance rates exceeding 77% among *Aeromonas* spp. isolates. Notably, among the quinolone resistance genes analyzed, only the *qnrS* gene was consistently detected. This observation agrees with the results of Marti et al. [46], who identified that 82.3% of CIP-resistant isolates were *Aeromonas* spp. harboring the *qnrS* gene. Although we did not measure antibiotic concentrations in our water samples, Martí et al. [46] reported CIP and sulfamethoxazole concentrations ranging from 50 to 70 and 12 to 30 ng/L, respectively, in the final effluent of the Ripoll WWTP (Spain). Although these concentrations are below the minimum inhibitory concentration (MIC), they may still promote AMR by altering gene expression, inducing stress responses, and facilitating HGT, thereby influencing microbial community dynamics [47].

*Aeromonas* spp. are known for their intrinsic resistance to β-lactams, including ampicillin, and first- [48] and second-generation [49] cephalosporins, due to chromosomally encoded, inducible AmpC-type β-lactamases. They may also acquire plasmid-mediated β-lactamase genes that further enhance resistance, which is of critical importance to human health [32]. In our study, over 50% of isolates were non-susceptible to FOX, TZP, and CTX, comparable with previous wastewater reports [28,50]. High TZP resistance can be attributed to the aforementioned AmpC-type β-lactamases, which reduce the efficacy of inhibitors such as TZP [13]. The high non-susceptibility to FOX and CTX observed could be associated with the high prevalence of *bla*_MOX_ among the β-lactam-resistant *Aeromonas* spp. isolates [49], consistent with the findings of Drk et al. [45] in municipal and hospital wastewater in Croatia.

In *Aeromonas* spp., carbapenem resistance is usually mediated by the chromosomal *cphA* gene or through the acquisition of clinically relevant plasmid-associated carbapenemase genes [15]. Although low non-susceptibility rates to IMI and MEM were observed, the detection of the *cphA*, *bla*_KPC_ and *bla*_VIM_ genes among the isolates studied highlights the occurrence of clinically relevant carbapenemase genes in environmental *Aeromonas* isolates [51].

Limited treatment options for MDR microorganisms have led to increasing use of COL. In recent decades, COL resistance has disseminated globally among bacteria from humans and animals, with plasmid-borne *mcr* genes reported across multiple countries. For this reason, COL use has been restricted or forbidden in food-producing animals in many regions and is now reserved as a last-resort treatment for MDR infections [52]. Although observed at low prevalence, COL resistance and the *mcr* genes were identified in MDR and XDR *Aeromonas* spp. in the present study, highlighting the concerning nature of their AMR profiles. Regarding the discrepancies observed between PCR-based detection and WGS, similar phenomena have been reported by Li et al. [53], where isolates showed phenotypic resistance to COL, but the corresponding genes were not detected by PCR. These differences may be explained by reduced gene expression, regulatory mechanisms, or gene silencing, which prevent observable resistance under laboratory conditions. In contrast, in our study, WGS allowed the detection of these silent or low-expression gene variants.

β-lactam and sulfonamide ARGs were detected throughout the WWTP treatment stages, including points at which treated water is reused and at the DWTP inlet. These data, together with previous reports [54,55], endorse sulfonamide- and β-lactamase-related genes as robust sentinels for monitoring AMR dissemination in wastewater systems.

HMTGs such as *merA* and *arsA* were widely detected. The *merA* and the *arsA* genes persisted across WWTP treatment stages, whereas only *merA* was present at the DWTP inlet. Our results are consistent with those of Luiza et al. [56], who found *merA* to be the most frequently detected HMTG, with *arsA* additionally observed among *Aeromonas* isolated from an urban estuary in Brazil. Notably, the *merA* gene is often located within class 1 integrons, which favors HGT [15]. In line with this, our data revealed a statistically significant correlation between *merA* and *intI1*. The study by Piotrowska et al. [15] also emphasized that co-localization of HMTGs with ARGs on a single MGE represents an important mechanism for co-selection. Our statistical analysis suggested a possible association between *merA* and *arsA* with specific ARGs, implying possible co-selection mechanisms.

Nearly 90% of isolates carried at least one VFG, highlighting the pathogenic potential of environmental *Aeromonas* isolates. As this genus is widely distributed in water, food, and soil [57], exposure through contaminated water or food may represent a potential route of infection [58]. For this reason, it is essential to identify pathogenic environmental *Aeromonas* spp. isolates that may pose a risk to human health [59]. Our results are consistent with studies on *Aeromonas* by Igbinosa et al. [60], who detected the *aerA*, *fla* and *hlyA* genes in wastewater samples from South Africa, and by Robertson et al. [61], who reported the *act* and *alt* genes in source and drinking water samples from Alabama. Our statistical analyses revealed a positive association between the *aerA* and *bla*_CPHA_ genes, whereas Govender et al. [21] reported a correlation between the *aerA* and the *bla*_OXA_ genes in *Aeromonas*. Whereas their study also found the *fla* gene to be significantly associated with the *sul1* and *sul2* genes, we found a significant association between the *fla* and *bla*_CTX-M-1_ genes.

In our study, biofilm-forming isolates were commonly detected in reclaimed water. Biofilms act as ecological niches that facilitate close cell-to-cell interactions and may reduce the efficiency of disinfection procedures such as chlorination [22,62]. Consequently, these isolates may survive treatment processes and re-emerge under favorable conditions.

HGT contributes significantly to the rapid dissemination of AMR, facilitating the spread of ARGs and the emergence of MDR isolates [23,63]. Antibiotic selective pressure, as reported by Martí et al. [46], promotes the horizontal acquisition of ARGs, fostering genome diversification and enhancing bacterial fitness [64]. Our conjugation experiments demonstrated horizontal transfer of resistance determinants associated with plasmid replicon types from *A. caviae* to *E. coli* and from *E. coli* to *A. caviae*, among other bacteria. Both donors were MDR, SBF, and carried the *intI1* gene, confirming plausible pathways for gene circulation. Not only was the transfer of ARGs from *A. caviae* to *E. coli* demonstrated, but also the transfer of VFGs, as reported by Mangar et al. [65].

Previous studies have linked the transfer of plasmid types such as IncU [66], ColE and IncN [45] from *Aeromonas* spp. to *E. coli*, but conjugation with *Aeromonas* spp. as recipients remains poorly explored. Matsushita et al. [67] demonstrated the transfer of the *bla*_IMP_ gene from *E. coli* to *A. caviae* via vesicle accumulation in the ciliate *Tetrahymena* sp., involving an IncA/C-type plasmid. Here, we demonstrated HGT from *E. coli* to *A. caviae*, including the transfer of *bla*_KPC_, detected in transconjugants carrying FrepB and ColE + IncU replicon types. Plasmid incompatibility types were identified by PCR-based replicon typing; therefore, structural linkage between specific ARGs and individual plasmid backbones was not resolved. Although transfer frequency was low (10^−8^), high bacterial densities and biofilm-mediated cell-to-cell proximity in WWTPs make even rare transfer events ecologically significant.

This issue is particularly relevant in the context of water reuse regulation. In Spain, Royal Decree 1085/2024 [68], in line with EU Regulation 2020/741 [69], allows reclaimed water to be applied in a wider range of municipal, industrial, and ecological applications. Within this regulatory scheme, *E. coli* serves as the primary microbiological indicator, while additional microbial criteria are only mandated under specific high-risk conditions. Nevertheless, *E. coli* is not an indigenous member of aquatic ecosystems; its detection in water bodies generally signals recent fecal contamination rather than the presence of a persistent aquatic community [70]. Therefore, in contexts where fecal indicators may be absent or limited, the inclusion of alternative microbial indicators such as *Aeromonas* could offer a more complete perspective on the emergence, maintenance, and environmental dissemination of AMR.

Our study has several limitations that should be considered when interpreting the findings. As it represents a case study conducted in a single metropolitan area, the results may not be directly generalizable to other geographic regions or water treatment systems with different environmental or operational conditions. In addition, the reliance on culture-based methods and MALDI-TOF identification may underestimate the diversity of *Aeromonas* populations present in these environments. The environmental context was not quantitatively characterized through measurements such as bacterial loads, physicochemical parameters, or antibiotic concentrations, which would have allowed a more comprehensive assessment of AMR dynamics across treatment stages. Furthermore, genomic analyses based on short-read sequencing and PCR-based plasmid typing limited the ability to fully resolve plasmid structures and determine the precise localization of resistance genes. Finally, the statistical analysis was designed to explore potential associations between genetic determinants, with results interpreted based on ORs and their confidence intervals as estimates of the strength and probability of these associations.

Future studies integrating quantitative environmental measurements, culture-independent approaches, long-read sequencing, and broader geographic sampling will be important to further elucidate the ecological role of *Aeromonas* spp. in AMR dissemination across urban water systems.

In conclusion, this study demonstrates that *Aeromonas* spp. remain detectable across multiple stages of WWTPs and DWTPs while carrying multidrug resistance, VFGs, HMTGs, and MGEs that facilitate HGT. These findings underscore their ecological and public health relevance, particularly in the context of reclaimed water reuse, and support their use as sentinel organisms for AMR monitoring and assessment of treatment efficacy, complementing traditional fecal indicators.

## 4. Materials and Methods

### 4.1. Wastewater and Drinking Water Sampling and Processing

Four sampling campaigns were carried out in the Baix Llobregat area of Barcelona, Spain, during the winter (30 January and 21 March) and summer (26 June and 25 July) of 2023. Samples were collected from the Baix Llobregat WWTP, located in the lower basin of the Llobregat River, and from the Gavà-Viladecans WWTP, which lies in the Llobregat Delta corridor. Additional samples were taken from the Sant Joan Despí, also located in the lower basin, whose intake is influenced by the reclaimed water of Baix Llobregat WWTP. The volume of collected water, preserved and processed per sampling point, as well as transport conditions such as time between sampling and processing and temperature during transport are described in the methodology outlined by Ballén et al. [25].

### 4.2. Isolation and Identification of Antibiotic-Resistant Aeromonas spp.

Isolation, selection and preservation were carried out as described [25]. Species identification was performed using matrix-assisted laser desorption ionization-time of flight mass spectrometry (MALDI Biotyper, Bruker Daltonics GmbH, Bremen, Germany). The database for identification was the reference MBT Compass Library DB-7854 (Bruker Daltonik GmbH, Bremen, Germany). The modified score values suggested by the manufacturer were used: A score ≥ 2.3 meant species identification; a score between 2.0 and 2.299 meant genus identification and probable species identification; a score between 1.7 and 1.9 meant probable genus identification; and a score < 1.69 meant non-reliable identification.

### 4.3. Antimicrobial Susceptibility Testing

The antimicrobial susceptibility profiles of the isolates were evaluated using the Kirby–Bauer disk diffusion method using Mueller–Hinton II agar (MHA) plates (Becton Dickinson (BD), Franklin Lakes, NJ, USA) and following the guidelines of the Clinical Laboratory Standards Institute (CLSI) [71]. The BD BBL™ Sensi-Disc™ (Becton Dickinson (BD), Franklin Lakes, NJ, USA) tested included CIP (5 µg), CHL (30 µg), GM (10 µg), MEM (10 µg), SXT (1.25/23.75 µg), TET (30 µg), AN (30 µg), TZP (100/10 µg), FEP (30 µg), CTX (30 µg), FOX (30 µg), CAZ (0 µg) and IMI (10 µg). *E. coli* ATCC 25922 was used as quality control.

To date, CLSI [72] and European Committee on Antimicrobial Susceptibility Testing guidelines (EUCAST) [73] recommend determining COL susceptibility using broth microdilution, broth elution or agar diffusion methods. However, these guidelines apply primarily to *Enterobacteriaceae* and *Pseudomonas aeruginosa*, and no epidemiological cut-off values (CO_WT_) have been established for other Gram-negative bacilli such as *Aeromonas* spp. [74]. Lin et al. [75] proposed a MIC CO_WT_ of 4 µg/mL for *Aeromonas* spp.

In addition, bacterial isolates were classified as non-susceptible to one or two antimicrobial categories, MDR, XDR, or pandrug-resistant (PDR). Following the approach of Zhou et al. [76], we adapted the *Enterobacteriaceae* criteria proposed by Magiorakos et al. [77]. Although these definitions were originally developed for *Enterobacteriaceae*, they are widely applied in studies of non-enterobacterial Gram-negative bacteria, including *Aeromonas* spp., to allow standardized comparison of resistance profiles. Intrinsic resistance traits of *Aeromonas* were considered when interpreting the resulting classifications.

Accordingly, MDR isolates were defined as isolates non-susceptible to at least one agent in three or more antimicrobial categories, XDR isolates as non-susceptible to at least one agent in all but two or fewer antimicrobial categories, and PDR isolates as non-susceptible to all agents in all antimicrobial categories [76].

### 4.4. Phenotypic Identification of Extended-Spectrum β-Lactamase (ESBL) and AmpC-Producing Isolates

For the phenotypic determination of ESBL and AmpC production, isolates non-susceptible to 3GC were tested. As CLSI guidelines [72] are not available for *Aeromonas* spp., we interpreted the results according to the CLSI guidelines for Enterobacterales, as previously applied by Bisi-Johnson et al. [78] and Drk et al. [45], respectively.

For ESBL production, bacterial cultures were standardized to a 0.5 McFarland turbidity and evenly spread on MHA to create a uniform lawn. An antibiotic disk containing amoxicillin/clavulanic acid (AMC) (20/10 μg) was positioned at the center of the plate, while two 3GC disks (CTX and CAZ) were placed symmetrically at a distance of either 15 mm or 20 mm (center-to-center) from the AMC disk. The plates were incubated at 37 °C for 24 h in an inverted position. ESBL production was indicated by a visible distortion or enlargement of the inhibition zone extending towards the AMC disk [78]. Quality control was performed using *E. coli* ATCC 25922.

The isolates phenotypically identified as ESBL producers were tested for the CTX-M group enzymes using the NG-Test^®^ CTX-M Multi (NG-Biotech Laboratories, Guipry-Messac, France). This assay detects enzymes from the five major CTX-M groups but does not differentiate the specific variant present.

The AmpC production was evaluated by placing one disk with CTX (30 µg) and another with CAZ (30 µg) at a distance of 20 mm (center to center) from a disk with phenyl boronic acid (PBA) (400 µg) and another with cloxacillin (CLX) (500 µg). An isolate was considered an AmpC producer if there was an extension of the inhibition halo of CTX or CAZ in the area near the disk with PBA or CLX [79].

### 4.5. Phenotypic Detection of Carbapenemase-Producing Isolates

Isolates showing non-susceptibility to MEM and IMI were further analyzed. Briefly, two 1 μL loopfuls of bacterial growth from an overnight blood agar culture were suspended in 2 mL of trypticase soy broth (TSB) (Condalab, Madrid, Spain) for the mCIM or in 2 mL of TSB containing 20 μL of 0.5 M EDTA for the EDTA-carbapenem inactivation method (eCIM). A 10 μg MEM disk (BD, Franklin Lakes, NJ, USA) was added to each suspension and incubated at 37 °C for 4 h. An MHA plate was prepared by inoculating it with a 0.5 McFarland standard suspension of *E. coli* ATCC 25922 as the indicator strain. After incubation, the MEM disks were removed from the TSB and TSB-EDTA suspensions and placed on the MHA plate inoculated with the ATCC strain. The plates were then incubated overnight at 37 °C. Zones of inhibition around the disks were measured. In the absence of CLSI [72] guidelines for this test in *Aeromonas* spp., the protocol for Enterobacterales was used as a reference, as suggested by Wang et al. [80]. Quality control was performed using *E. coli* ATCC 25922.

Isolates yielding a positive mCIM were further analyzed with the NG-Test^®^ DetecTool CARBA 5 (NG-Biotech Laboratories, Guipry-Messac, France) for the detection of the five main carbapenemase enzymes: KPC, OXA-48-like, VIM, IMP, and NDM.

### 4.6. Detection of Antibiotic Resistance, Integrase, Heavy Metal Tolerance and Virulence Factor Genes

A colony from an overnight culture of each isolate was selected and suspended in 200 μL of Milli-Q water. The suspension underwent a 10 min boiling step, followed by centrifugation at 13,000 rpm for 10 min. The supernatant obtained was then used as the DNA template for PCR analysis. The primers used to analyze the different ARGs, integrase genes, HMTGs and VFGs are listed in Supplementary Material Table S1.

### 4.7. Biofilm Formation and Quantification

The assay was performed based on the protocol used by El-Hossary et al. [81] with slight modifications. Briefly, *Aeromonas* spp. isolates were initially grown on Columbia agar supplemented with 5% sheep blood at 37 °C for 18–24 h. The resulting colonies were inoculated into TSB and incubated at 37 °C for 24 h. Cultures were then diluted 1:100 in fresh TSB and transferred to 96-well polystyrene plates (Nunc™ Edge 2.0, VWR International, Radnor, PA, USA), followed by static incubation for 48 h at 37 °C. Control wells contained sterile TSB, without bacterial inoculation. Post-incubation, wells were carefully rinsed with phosphate-buffered saline (PBS) 1×, air-dried, and stained with 2% (*v/v*) crystal violet for 10 min at room temperature. The wells were rinsed with PBS 1×, and after drying, the biofilm-bound stain was dissolved in 33% glacial acetic acid. The biofilm biomass was quantified by measuring OD at 580_nm_ with a microplate reader (EPOCH 2 microplate reader; BioTek, Winooski, VT, USA). The experiment was carried out in three technical and biological replicates. The OD results were interpreted following the criteria established by Stepanovic et al. [82]. The biofilm formation capability of *Aeromonas* spp. isolates was expressed as follows: NBF (OD ≤ 0.204), WBF (0.204 ≤ OD ≤ 0.408), MBF (0.408 ≤ OD ≤ 0.817), and SBF (OD ≥ 0.817). Culture medium without inoculum was used as a sterility control. Experimental readings were normalized against this negative control to determine cut-off values, ensuring methodological consistency.

### 4.8. Conjugation

To evaluate the capacity for HGT, two in vitro conjugation assays were conducted using a standardized protocol in our laboratory. The first assay aimed to assess the transfer of ARGs via plasmids from *E. coli* to *Aeromonas* spp.

Sixteen *Aeromonas* spp. isolates carrying ESBL genes were used as plasmid donors, while *E. coli* isolate CV601, resistant to rifampin (RIF), was used as the recipient. Donor and recipient isolates were grown overnight at 37 °C and 140 rpm in Luria Broth (LB) (Condalab, Madrid, Spain) supplemented with CTX (50 µg/mL) and RIF (50 µg/mL), respectively. OD at 600_nm_ was measured and adjusted to 0.5 for all cultures. For each mating pair, 750 µL of donor and 750 µL of the recipient were mixed, centrifuged for 10 min at 5000 rpm, and resuspended in 100 µL of LB. The mixture was spotted onto a MF-Millipore™ membrane filter (0.45 µm pore size, 25 mm diameter; Merck Millipore, Burlington, MA, USA) for mating and incubated overnight at 37 °C. Filters were then washed in LB, and 100 µL of the mixture was spread on LB agar plates containing RIF (50 µg/mL), CTX (50 µg/mL), or both (RIF + CTX 50 µg/mL each) and incubated overnight at 37 °C.

The second conjugation assay evaluated gene transfer in the opposite direction, from *Aeromonas* spp. to *E. coli*. Four *E. coli* isolates from our previous study [25] carrying carbapenemase genes were used as donors, and one *Aeromonas* spp. isolate harboring *cmlA* served as the recipient. The procedure was identical to the first assay, except that the antibiotics used were MEM (4 µg/mL) for the donor and CHL (32 µg/mL) for the recipient.

Transfer frequencies were calculated as the total number of transconjugants divided by the total number of recipients [83]. Transconjugants were subjected to PCR analysis to verify the successful transfer of the targeted ARGs. To further confirm that the transconjugants retained a genetic fingerprint consistent with the recipient strain, repetitive extragenic palindromic PCR was performed, and the primers used were REP-F (5′-IIIGCGCCGICATCAGGC-3′) and REP-R (5′-ACGTCTTATCAGGCCTAC-3′) [84].

### 4.9. PCR-Based Replicon Typing

This technique was used to identify the replicon type of plasmids in isolates with successful conjugation. PCR amplification was carried out using DNA from donor and transconjugant isolates with the specific primer sets listed in Table S2.

### 4.10. Sequencing, Annotation and Phylogenomic Analysis

The DNA was extracted using the MagMAX™ DNA Multi-Sample Ultra 2.0 Kit in a KingFisher Flex (Thermo Fisher Scientific, Waltham, MA, USA) and quantified with a Qubit Flex Fluorometer (Thermo Fisher Scientific). Genomic libraries were prepared using the DNA Prep Library Preparation Kit, followed by paired-end sequencing (2 × 150) on a NextSeq platform (Illumina, San Diego, CA, USA).

Quality assessment and genome assembly were carried out using the Bactopia pipeline (https://github.com/bactopia/bactopia) (accessed on 6 November 2025) [85], which includes read quality control with FastQC (www.bioinformatics.babraham.ac.uk/projects/fastqc/) (accessed on 6 November 2025) [86], genome assembly with Shovill (https://github.com/tseemann/shovill) (accessed on 9 September 2025) [87], annotation with Prokka (https://github.com/tseemann/prokka) (accessed on 9 September 2025) [88], sequence typing using the *Aeromonas* spp. MLST scheme from the PubMLST (https://pubmlst.org/organisms/aeromonas-spp) (accessed on 9 September 2025) [89], and detection of acquired ARGs by AMRFinder+ (https://github.com/ncbi/amr) (accessed on 6 November 2025) [90]. *Aeromonas* spp. identification was conducted with rMLST (https://pubmlst.org/species-id) (accessed on September 2025) [91]. The phylogenetic tree was constructed based on a pangenome analysis with Roary (https://sanger-pathogens.github.io/Roary/) (accessed on 6 November 2025) [92] with a minimum percentage of blastp identity of 80% and without split paralogs. The 1250 genes present in all genomes were used for tree reconstruction with IQ-tree (https://iqtree.github.io/) (accessed on 6 November 2025) GTR + F+I + G4 model [93] and 1000 UFBoot replicates. Final tree and metadata were visualized with iTOL (https://itol.embl.de/) (accessed on 6 November 2025) [94]. Reads were deposited at the European Nucleotide Archive (PRJEB102387).

### 4.11. Statistical Analyses

Associations between ARGs, integrase genes, HMTGs, VFGs, and biofilm formation were evaluated. All variables were categorical. Bivariate associations were assessed using the Pearson’s χ^2^ or Fisher’s exact test when expected counts were low, with effect size quantified by Cramer’s V [95]. Univariable logistic regression models were fitted using Firth’s penalized likelihood correction to reduce bias from complete separation or sparse data [96]. Analyses were performed in Stata 19 (StataCorp, College Station, TX, USA, 2025).

## Figures and Tables

**Table 1:** Number of *Aeromonas* spp. isolates harboring the *merA* and *arsA* genes at different treatment stages in the Baix Llobregat WWTP, the Gavà-Viladecans WWTP, and the Sant Joan Despí DWTP. DWTP: drinking water treatment plant, WWTP: wastewater treatment plant.

		*merA*	*arsA*
Baix Llobregat WWTP	Primary inlet	20	2
	Secondary outlet	14	1
	Tertiary outlet	19	1
	Advanced tertiary outlet	4	0
Gavà-Viladecans WWTP	Primary inlet	16	2
	IFAS Secondary outlet	12	1
	MBR Secondary outlet	11	1
	Tertiary outlet	3	0
Sant Joan Despí DWTP	Inlet	2	0
	Outlet	0	0

**Table 2:** Characteristics of donors, recipients, and transconjugants: genes harbored and transferred, and plasmid incompatibility groups.

Donor	Donor Plasmid Replicons Detected	Harbored Genes	Recipient	Transconjugant Plasmid Replicons Detected	Transferred Genes
*A. caviae* 123	IncW	*bla*_PER_, *bla*_MOX_, *bla*_OXA-1161_, *sul1*, *intI1*, *act*, *alt*	*E. coli* CV601	IncW	*bla*_PER_, *sul1*, *intI1*, *act*
*E. coli* 1362	FrepB, ColE, IncU	*bla*_KPC_, *bla*_EC_, *bla*_OXA-1_, *mph(A)*, *arr-3*, *sul1*, *qnrS*, *aph(3′)-la*, *aac(6′)-lb-cr5*, *catB3*, *merA*, *arsA*, *intI1*	*A. caviae* 214	(FrepB), (ColE + IncU)	*bla*_KPC_

## Data Availability

The original data presented in the study are openly available in the European Nucleotide Archive (ENA) under BioProject accession number PRJEB102387.

## Supplementary Materials

The following supporting information can be downloaded at: https://www.mdpi.com/article/10.3390/antibiotics15030301/s1, Figure S1. SNP-based phylogenetic analysis of ST3458 isolates. Table S1. Primers used for antibiotic resistance, integrase, heavy metal tolerance and virulence factor genes detection by PCR. Table S2. Specific primers and conditions used in plasmid replicon typing. References [97,98,99,100,101,102,103,104,105,106,107,108,109,110,111,112,113,114,115] are cited in the Supplementary Materials.
