# Supplementary material for: Characterizing Aeromonas spp. as a Potential Sentinel Organism for Antimicrobial Resistance Dissemination in Wastewater and Drinking Water Treatment Systems: A Case Study in the Barcelona Metropolitan Area, Spain
Source: Antibiotics (Basel). 2026 Mar 17;15(3):301. doi: 10.3390/antibiotics15030301 (PMC13024261; doi:10.3390/antibiotics15030301)
Supplement: Supplementary file 1 [file antibiotics-15-00301-s001.zip › antibiotics-4193311-supplementary.pdf]

Supplementary Material:

Figure S1. SNP-based phylogenetic analysis of ST3458 isolates.

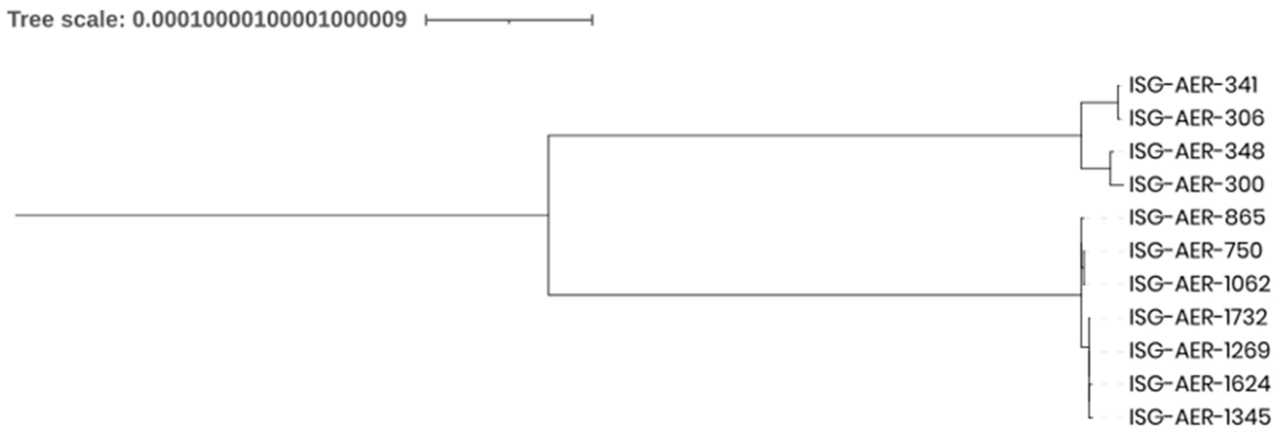

Table S1. Primers used for antibiotic resistance, integrase, heavy metal tolerance and virulence factor genes detection by PCR.

		Primer name	Sequence (5′->3′)	Product size (bp)	Annealing temp. (°C)	Reference
Antibiotic resistance genes	β-lactams	bla_{MOX}-F	GCTGCTCAAGGAGCACAGGAT	520	64	[97]
		bla_{MOX}-R	CACATTGACATAGGTGTGGTGC			
		bla_{CIT}-F	TGGCCAGAACTGACAGGCAAA	462	64	[97]
		bla_{CIT}-R	TTTCTCCTGAACGTGGCTGGC			
		bla_{DHA}-F	AACTTTCACAGGTGTGCTGGGT	405	64	[97]
		bla_{DHA}-R	CCGTACGCATACTGGCTTTGC			
		bla_{EBC}-F	TCGGTAAAGCCGATGTTGCGG	302	64	[97]
		bla_{EBC}-R	CTTCCACTGCGGCTGCCAGTT			
		bla_{FOX}-F	AACATGGGGTATCAGGGAGATG	190	64	[97]
		bla_{FOX}-R	CAAAGCGCGTAACCGGATTGG			
		bla_{TEM}-F	TCGCCGCATACACTATTCTCAGAATGA	445	50	[98]
		bla_{TEM}-R	ACGCTCACCGGCTCCAGATTTAT			
		bla_{SHV}-F	ATGCGTTATATTCGCCTGTG	747	56	[98]
		bla_{SHV}-R	TGCTTTGTTATTCGGGCCAA			
		bla_{GES}-F	AGTCGGCTAGACCGGAAAG	399	60	[99]
		bla_{GES}-R	TTTGTCCGTGCTCAGGAT			
		bla_{PER}-F	GCTCCGATAATGAAAGCGT	520	60	[99]
		bla_{PER}-R	TTCGGCTTGACTCGGCTGA			
		bla_{VEB}-F	CATTTCCCGATGCAAAGCGT	648	60	[99]
		bla_{VEB}-R	CGAAGTTTCTTTGGACTCTG			
		$bla_{CTX-M-1}$-F	AAAAATCACTGCGCCAGTTC	415	52	[100]
		$bla_{CTX-M-1}$-R	AGCTTATTCATCGCCACGTT			
		$bla_{CTX-M-9}$-F	CAAAGAGAGTGCAACGGATG	205	52	[100]
		$bla_{CTX-M-9}$-R	ATTGGAAAGCGTTCATCACC			
		cphA-F	GCTTAGAGCTCCTAAGGAGCAAGATGAAAGGTTGG	720	53	[81]
		cphA-R	GCATAGGTACCTTATGACTGGGGTGCGGCCTTG			
		bla_{KPC}-F	TCGCCGTCTAGTTCTGCTGTCTTG	353	66	[101]
		bla_{KPC}-R	ACAGCTCCGCCACCGTCAT			
	Tetracyclines	*tetA*-F	GCTACATCCTGCTTGCCTTC	210	55	[102]

	tetA-R	CATAGATCGCCGTGAAGAGG			
	tetB-F	TTGGTTAGGGGCAAGTTTTG	659	55	[102]
	tetB-R	GTAATGGGCCAATAACACCG			
Sulfonamides	*sul1-F*	CTTCGATGAGAGCCGGCGGC	436	65	[103]
	sul1-R	GCAAGGCGGAAACCCGCGCC			
	sul2-F	TCAACATAACCTCGGACAGT	707	55	[103]
	sul2-R	GATGAAGTCAGCTCCACCT			
Aminoglycosides	*aph(6)-Id*-F	GACTCCTGCAATCGTCAAGG	560	55	[104]
	aph(6)-Id-R	GCAATGCGTCTAGGATCGAG			
	aph(3″)-Ib-F	GTGGCTTGCCCCGAGGTCATCA	612	55	[105]
	aph(3″)-Ib-R	CCAAGTCAGAGGGTCCAATC			
	aadA5-F	CGCTCAACGCAAGATTCTCT	792	55	[106]
	aadA5-R	ATGGGTGAATTTTTCCCTGCAC			
	acc(6′)-Ib-cr-F	TTGCGATGCTCTATGAGTGGCTA	482	55	[107]
	acc(6′)-Ib-cr-R	CTCGAATGCCTGGCGTGTTT			
Chloramphenicol	*catI*-F	AGTTGCTCAATGTACCTATAACC	547	55	[108]
	catI-R	TTGTAATTCATTAAGCATTCTGCC			
	cmIA-F	CCGCCACGGTGTTGTTGTTATC	698	55	[108]
	cmIA-R	CACCTTGCCTGCCCATCATTAG			
Quinolones	*qnrA*-F	ATTTCTCACGCCAGGATTTG	516	53	[109]
	qnrA-R	GATCGGCAAAGGTTAGGTCA			
	qnrB-F	GATCGTGAAAGCCAGAAAGC	469	53	[110]
	qnrB-R	ACGATGCCTGGTAGTTGTCC			
	qnrC-F	GGGTTGTACATTTATTGAATC	447	47	[111]
	qnrC-R	TCCACTTTACGAGGTTCT			
	qnrS-F	ACGACATTCGTCAACTGCAA	417	53	[109]
	qnrS-R	TAAATTGGCACCCTGTAGGC			
Colistin	*mcr-3-F*	GCTGTATCCGTTCCCTGCAT	511	60	This paper
	mcr-3-R	CGTAGAGCAATGCGGTGTTG			
Silver	*silA*-F	GCAAGACCGGTAAAGCAGAG	936	59	[112]

Heavy metal tolerance genes		*silA*-R	CCTGCCAGTACAGGAACCAT			
	Mercury	*merA*-F	ACCATCGGCGGCACCTGCGT	1238	63	[113]
		merA-R	ACCATCGTCAGGTAGGGGAACA			
	Copper	*pcoD*-F	CAGGAACGGTGATTGTTGTA	700	55	[112]
		pcoD-R	CCGTAAAATCAAAGGGCTTA			
	Arsenic	*arsA*-F	ACTGGCTTACTGACTGACGC	560	60	This paper
		arsA-R	GGCTTGTTCTGTGGGCTTTG			
Virulence factor genes	Aerolysin	*aerA*-F	CCTATGGCCTGAGCGAGAAG	431	64	[61]
		aerA-R	CCAGTTCCAGTCCCACCACT			
	Cytotoxic enterotoxin	*act*-F	TACCACCACCTCCCTGTCGC	249	60	[113]
		act-R	ATGCTGCTCGCCTTGTGGTT			
	Flagellin	*fla*-F	TCCAACCGTYTGACCTC	608	54	[61]
		fla-R	GMYTGGTTGCGRATGGT			
	Heat-labile cytotonic enterotoxin	*alt*-F	CCATCCCCAGCCTTTACGCCAT	338	63	[61]
		alt-R	TTTCACCGAGGTGACGCCGT			
	Haemolysin	*hlyA*-F	GGCCGGTGGCCCGAAGATACGGG	597	62	[61]
		hlyA-R	GGCGGCGCCGGACGAGACGGG			

Table S2. Specific primers and conditions used in plasmid replicon typing.

Replicon	Primer name	Sequence (5′->3′)	Product size (bp)	Annealing temperature (°C)	Reference
HI1	HI1-F	GGAGCGATGGATTACTTCAGTAC			
	HI1-R	TGCCGTTTCACCTCGTGAGTA	471		
HI2	HI2-F	TTTCTCCTGAGTCACCTGTTAACAC			
	HI2-R	GGCTCACTACCGTTGTCATCCT	644		
I1-Iγ	I1-F	CGAAAGCCGGACGGCAGAA			
	I1-R	TCGTCGTTCCGCCAAGTTCGT	139		
X	X-F	AACCTTAGAGGCTATTTAAGTTGCTGAT			
	X-R	TGAGAGTCAATTTTTATCTCATGTTTTAGC	376		
L/M	L/M-F	GGATGAAAACTATCAGCATCTGAAG			
	L/M-R	CTGCAGGGGCGATTCTTTAGG	785		
N	N-F	GTCTAACGAGCTTACCGAAG			

	N-R	GTTTCAACTCTGCCAAGTTC	559		
FIA	FIA-F	CCATGCTGGTTCTAGAGAAGGTG			
	FIA-R	GTATATCCTTACTGGCTTCCGCAG	462		
FIB	FIB-F	GGAGTTCTGACACACGATTTTCTG			
	FIB-R	CTCCCGTCGCTTCAGGGCATT	702		
W	W-F	CCTAAGAACAACAAAGCCCCCG			
	W-R	GGTGCGCGGCATAGAACCGT	242		
Y	Y-F	AATTCAAACAACACTGTGCAGCCTG			
	Y-R	GCGAGAATGGACGATTACAAAACTTT	765		
P	P-F	CTATGGCCCTGCAAACGCGCCAGAAA			
	P-R	TCACGCGCCAGGGCGCAGCC	534	60	[114]
FIC	FIC-F	GTGAACTGGCAGATGAGGAAGG			
	FIC-R	TTCTCCTCGTCGCCAAACTAGAT	262		
A/C	A/C-F	GAGAACCAAAGACAAAGACCTGGA			
	A/C-R	ACGACAAACCTGAATTGCCTCCTT	465		
T	T-F	TTGGCCTGTTTGTGCCTAAACCAT	750		
	T-R	CGTTGATTACACTTAGCTTTGGAC			
FIIs	FIIs-F	CTGTCGTAAGCTGATGGC			
	FIIs-R	CTCTGCCACAAACTTCAGC	270		
K	K/B-F	GCGGTCCGGAAAGCCAGAAAAC			
	K-R	TCTTTCACGAGCCCGCCAAA	160		
B/O	K/B-F	GCGGTCCGGAAAGCCAGAAAAC		60	
	B/O-R	TCTGCGTTCCGCCAAGTTCGA	159		[114]
FrepB	FrepB-F	TGATCGTTTAAGGAATTTTG			
	FrepB-R	GAAGATCAGTCACACCATCC	270	52	
ColE	oricolE-F	GTTCGTGCATACAGTCCA			
	oricolE-R	GGCGAAACCCGACAGGACT	187	57	
R	IncR-F	TCGCTTCATTCCTGCTTCAGC			
	IncR-R	GTGTGCTGTGGTTATGCCTCA	251		
U	InU-F	TCACGACACAAGCGCAAGGG			[115]
	IncU-R	TCATGGTACATCTGGGCGC	843	62	
